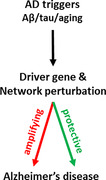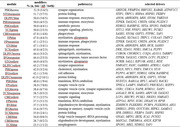# Systems genetic dissection of Alzheimer’s disease brain gene expression networks

**DOI:** 10.1002/alz.089491

**Published:** 2025-01-03

**Authors:** Pinghan Zhao, Omar El Fadel, Anh Le, Carl Grant Mangleburg, Timothy Wu, Justin Dhindsa, Bismark K Amoh, Aditi Sai Marella, Yarong Li, Nicholas T Seyfried, Allan I. Levey, Zhandong Liu, Ismael Al‐Ramahi, Juan Botas, Joshua M Shulman

**Affiliations:** ^1^ Jan and Dan Duncan Neurological Research Institute, Texas Children’s Hospital, Houston, TX USA; ^2^ Baylor College of Medicine, Houston, TX USA; ^3^ Baylor College of Medicine, Neuroscience Program, Houston, TX USA; ^4^ Jan and Dan Duncan Neurological Research Institute, Houston, TX USA; ^5^ Emory University Center for Neurodegenerative Disease, Atlanta, GA USA; ^6^ Emory University School of Medicine, Atlanta, GA USA; ^7^ Emory University, Atlanta, GA USA

## Abstract

**Background:**

In Alzheimer’s disease (AD), changes in the brain transcriptome are hypothesized to mediate the impact of neuropathology on cognition. Gene expression profiling from postmortem brain tissue is a promising approach to identify causal pathways; however, there are challenges to definitively resolve the upstream pathologic triggers along with the downstream consequences for AD clinical manifestations.

**Method:**

We have functionally dissected 30 AD‐associated gene coexpression modules using a cross‐species strategy in *Drosophila melanogaster* models. First, integrating longitudinal RNA‐sequencing and fly behavioral phenotyping, we interrogated unique and shared transcriptional responses to amyloid beta (Aβ) plaques, tau neurofibrillary tangles, and/or aging, along with potential links to progressive neuronal dysfunction. In order to confirm causal modules and pinpoint AD network drivers, we next performed systematic *in vivo* genetic manipulations of 357 conserved, prioritized targets to identify modifiers of Aβ‐ and/or tau‐induced neurodegeneration. We subsequently partitioned candidate causal subnetworks, which were further validated based on human or *Drosophila* genetic evidence.

**Result:**

Our results highlight hundreds of conserved, differentially expressed genes mapping to AD regulatory networks. Our systematic screening identified 144 tau/Aβ modifiers. We discovered an up‐regulated, causal network that is significantly enriched for both AD risk variants and markers of immunity / inflammation, and which promotes Aβ and tau‐mediated neurodegeneration based on fly genetic manipulations in neurons. By contrast, a promising synaptic regulatory network is strongly downregulated in human AD and is enriched for loss‐of‐function suppressors of Aβ/tau, consistent with a potential compensatory response to glutamatergic excitotoxic brain injury.

**Conclusion:**

our cross‐species, systems genetic approach establishes a putative causal chain linking AD pathology, large‐scale gene expression perturbations, and ultimately, neurodegeneration.